# Multi-block data integration analysis for identifying and validating targeted N-glycans as biomarkers for type II diabetes mellitus

**DOI:** 10.1038/s41598-022-15172-z

**Published:** 2022-06-29

**Authors:** Eric Adua, Ebenezer Afrifa-Yamoah, Emmanuel Peprah-Yamoah, Enoch Odame Anto, Emmanuel Acheampong, Kwaafo Akoto Awuah-Mensah, Wei Wang

**Affiliations:** 1 Rural Clinical School, Medicine and Health, University of New South Wales, Sydney, NSW Australia; 2 School of Medical and Health Sciences, Edith Cowan University, Joondalup, WA Australia; 3 School of Science, Edith Cowan University, Joondalup, WA Australia; 4 Department of Chemistry, East Tennessee State University, Johnson City, TN USA; 5 Department of Medical Diagnostics, Faculty of Allied Health Science, Kwame Nkrumah University of Science and Technology, 9800 Kumasi, Ashanti Region Ghana; 6 Department of Mathematics, University of Environment and Sustainable Development, Somanya, Ghana; 7 Centre for Precision Health, Edith Cowan University, Joondalup, Australia

**Keywords:** Biochemistry, Biomarkers, Diseases, Molecular medicine

## Abstract

Plasma N-glycan profiles have been shown to be defective in type II diabetes Mellitus (T2DM) and holds a promise to discovering biomarkers. The study comprised 232 T2DM patients and 219 healthy individuals. N-glycans were analysed by high-performance liquid chromatography. The multivariate integrative framework, DIABLO was employed for the statistical analysis. N-glycan groups (GPs 34, 32, 26, 31, 36 and 30) were significantly expressed in T2DM in component 1 and GPs 38 and 20 were related to T2DM in component 2. Four clusters were observed based on the correlation of the expressive signatures of the 39 N-glycans across T2DM and controls. Cluster A, B, C and D had 16, 16, 4 and 3 N-glycans respectively, of which 11, 8, 1 and 1 were found to express differently between controls and T2DM in a univariate analysis $(p < 0.05)$. Multi-block analysis revealed that trigalactosylated (G3), triantennary (TRIA), high branching (HB) and trisialylated (S3) expressed significantly highly in T2DM than healthy controls. A bipartite relevance network revealed that HB, monogalactosylated (G1) and G3 were central in the network and observed more connections, highlighting their importance in discriminating between T2DM and healthy controls. Investigation of these N-glycans can enhance the understanding of T2DM.

## Introduction

Type II diabetes mellitus (T2DM), characterised by persistent rise in plasma glucose^1,2^, may have existed over two centuries ago. At the time, the disease could kill within weeks or months of diagnosis^3^. After several decades, investments in T2DM research enhanced understanding of the condition, resulting in the development of treatments that improved quality of life and increased longevity^3^. Sadly, the path to cure the disease has been slow despite significant achievements and, in fact, the disease is still recognised as the fastest chronic condition that reduces the life expectancy of millions of people worldwide^3^.

It is a public knowledge that effective biomarkers can promote early detection, which in turn, can stimulate a timely intervention, and delay the onset of T2DM^2,4^. However, efforts to obtain robust biomarkers for the condition have been hindered by the complex nature of the condition. Indeed, the disease is the outcome of genetic, epigenetic, and environmental triggers, all of which complicates detection, diagnosis, and prediction^5^. Complex sugars, hereafter called glycans, represent an intermediate phenotype, linking our genetic architecture and the environment^5–7^. At the molecular level, glycans through the concerted actions of glycosidases and glycosyltransferases in the endoplasmic reticulum and Golgi apparatus, attach to proteins^5,7,8^ in a process termed glycosylation.

Amongst the multiple glycosylation types including O-linked, C-linked and S-linked, the present study focuses on N-glycan, which involves the binding of complex sugars to the asparagine residues of amino acids^8,9^. When complexed to proteins, N-glycans can change protein conformation, function and solubility^10,11^. Moreover, since their biosynthesis are largely influenced by the condition of the cell, profiling N-glycan signatures can allow for capturing the changes associated with pathophysiological state of the body^7,11,12^. Hence, the overwhelming evidence that N-glycan aberrations result in multiple diseases including cancers^13^, rheumatoid arthritis^14^, systemic lupus erythematosus^15^, hypertension^16^ as well as T2DM^5,17,18^.

The process of identifying N-glycan biomarkers depends heavily on sophisticated and high-throughput instruments including ultra-performance liquid chromatography (UPLC)^5^, mass spectrometry (MS)^19^, capillary electrophoresis (CE)^20,21^ and nuclear magnetic resonance imaging (NMR)^22^. These technologies are not only useful for the quantitative detection or measurement of traits in biological samples^8^, they also generate a global wealth of N-glycan data that are only interpretable with statistical methods. Examples of such methods include univariate analysis (e.g. ANOVA, t-tests), conventional multivariate analysis, logistic regression, or cox-regression methods^23^.

While these methods unravel defective biomarkers in diseased states, their biological interaction are not revealed in real time. This is partly because, the big data generated from complex technologies such as those mentioned poses formidable statistical modelling challenges including data over-fitting, curse of dimensionality, and multicollinearity^24,25^. It is even worsened with the N-glycan heterogeneity and variability of individual expression of glycans traits^5,7^. Moreover, for OMICS datasets, the number of variables exceeds the number of observations^26^.

Over the years, data integration methods such as Bayesian methods, network analysis, matrix factorisation methods and correlation-based analysis can circumvent some of these challenges and allow a more comprehensive and system level means of interrogating data. Moreover, such methods are suitable for interrogating any form of dataset, be it categorical, binary, or continuous. In addition, data integration addresses issues with missing data, systematic bias, and high error rates. The benefit of using integrative method is that it reduces the dimensions of global data, allows variables in complex data to be interrogated, enables the revelation of hidden structures, the determination of correlation trends, does not require a priori biological knowledge and the interpretation of trends in sample datasets. For example, dimension reduction techniques such as principal component analysis (PCA) permits changes of metabolites to be visualised^27^, to provide comprehensive insight into biological systems. However, a limitation of these standard methods is their failure to fully explore the connectivity of multiple networks^28,29^.

Recently, Singh et al.^30^ proposed the Data Integration Analysis for Biomarker discovery using Latent cOmponents (DIABLO) to reveal potential biomarkers from multi-omics assays. DIABLO is a supervised multi-omics method that simultaneously identifies key biomarkers in an integrated process by discriminating distinct groups. With this method, researchers have gained insight into the molecular patterns spanning across biological domains or characterizing certain phenotypes, and thus can identify multi-block biomarkers that are predictive of diseases. In a multivariate integrative framework, DIABLO uses a multi-step approach to concatenate all data, maximizes common information between multi-block datasets and applies a classification model to each block of the data. With powerful visualization capabilities, multiple phenotypes can be expressed in definitive plots to aid easy interpretation of the multiplicity of relationships in a multi-block dataset.

In this study, we attempt to assess the added value of DIABLO to holistically construct an integrated network that captures all possible of N-glycan-glycan interactions in T2DM and healthy controls. Understanding the interaction between N-glycan datasets can offer useful insights in glycan mechanisms.

## Methods and study design

**Figure 1:**
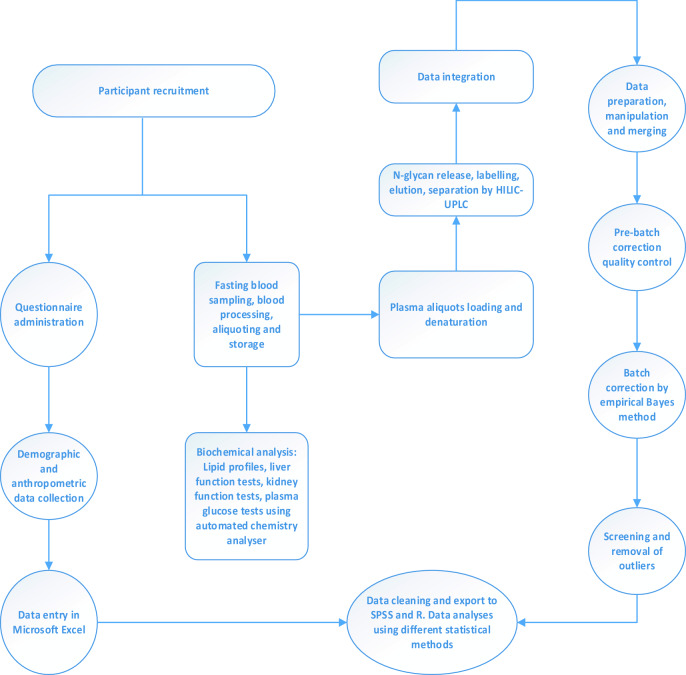
Flow chart of N-glycan data processing. Participants with no prior history of T2DM were recruited from the Kumasi metropolis. Ethics was approved and each participant was asked to complete a questionnaire. After this, demographic and anthropometric data were obtained, and fasting blood samples were collected for biochemical and N-glycan analysis. Statistical analyses were performed in SPSS and R.

In this cross-sectional study, we recruited 232 T2DM and 219 age-gender matched healthy controls. T2DM individuals were purposively sampled from the Diabetic Unit of the Komfo Anokye Teaching Hospital (KATH), Kumasi, Ghana whereas the controls were recruited by convenient sampling from three suburbs within the Kumasi metropolis (Fig. 1). The Committee on Human Research, Publication and Ethics (CHRPE) of Kwame Nkrumah University of Science and Technology (KNUST), Ghana, and the Human Research Ethics Committee (HREC) of Edith Cowan University (ECU) reviewed all aspects of the study and approved it. Written informed consent was obtained from all participants. All aspects of the study were conducted in consistence with the principles of the Helsinki’s Declaration.

### Inclusion criteria

T2DM was established based on the international classification of disease 10 (ICD-10) criteria and known history of anti-diabetes medication use. The controls, however, were individuals who were not suffering from T2DM and/or hypertension and had no history of anti-diabetes or antihypertensive medication use. In both groups, we excluded participants who were suffering from other chronic diseases related to the genitourinary, digestive, respiratory and haematological systems. The age range for all participants was 30–80 years.

### Anthropometric examination

Participants supplied their demographic information by completing a brief questionnaire after which anthropometric measurements including weight, height, Body mass index (BMI), Waist-to-hip ratio (WHR), Waist-to-height ratio (WHtR), systolic blood pressure (SBP) and diastolic blood pressure (DBP) were measured by standard methods (Fig. 1).

### Clinical data

Briefly, venous fasting blood samples were collected from each participant into tubes containing EDTA (ethylene diamine tetraacetic acid), fluoride oxalate and gel separator. Different clinical tests including Fasting plasma glucose (FPG), glycosylated haemoglobin (HbA1c), total cholesterol (TC), high density lipoprotein cholesterol (HDL-c), Low density lipoprotein cholesterol (LDL-c), triglycerides (TG) and very low-density lipoprotein cholesterol (VLDL-c) were measured on the automated chemistry analyser (Roche Diagnostics, COBAS INTEGRA 400 Plus, USA). WHtR was then calculated. Aliquots of processed plasma samples were stored at − 80 °C until N-glycan analysis (Fig. 1).

### N-glycan release and labelling

**Figure 2:**
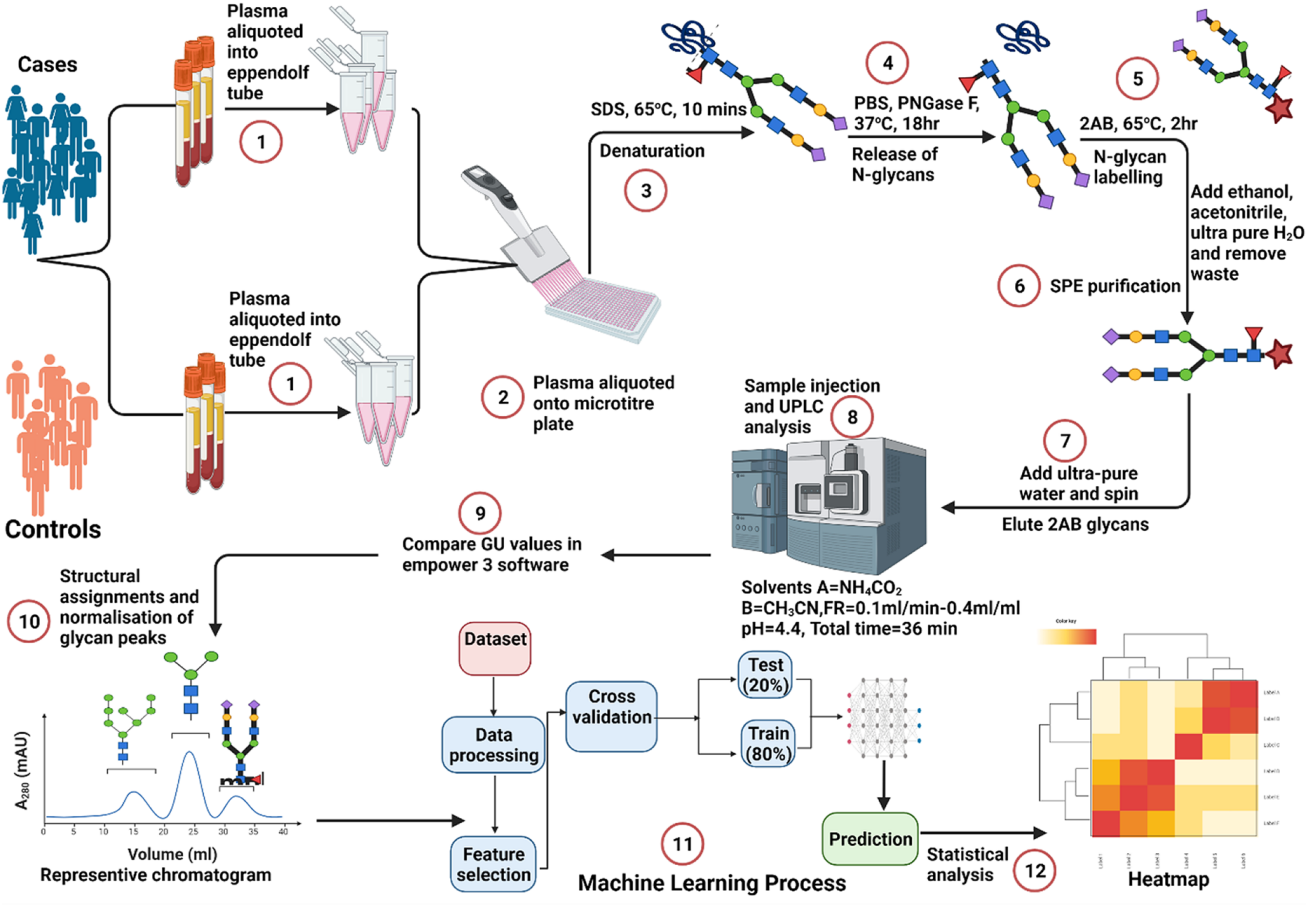
Workflow of N-glycan analysis with UPLC-FLR. Plasma samples were aliquoted into 96 well plates and denatured with sodium dodecyl sulphate (SDS). The plate was sealed and incubated at 65 °C for 10 min. IGEPAL CA-630 was added and sample mixed by pipetting up and down. This was then followed by incubation at room temperature. Glycans were freed from their bound glycoproteins by adding peptide N-glycosidase F (PN-Gase F) and incubation at 37 °C for 18 h. glycans were then fluorescently labelled with 2-aminobenzamide and incubated for 2 h at 65 °C. This was followed by four-step washing procedure with acetonitrile and 2AB glycans were eluted using ultra-pure water. Samples were injected into the UPLC and analysed under the following conditions: solvent A = 100 Mm ammonium formate, solvent B = acetonitrile, flow rate 0.1 ml/min, pH = 4.4. Structural assignments and normalisation of glycan peaks were then performed.

Plasma samples were first randomised on multiple plates to avoid bias and experimental errors. Plasma samples aliquoted in 96-well plates were denatured, following which, glycans were released, fluorescently labelled, purified/washed/cleaned up as described in our previous studies^5,31,32^. Hydrophilic interaction liquid chromatography on a Waters Acquity ultra-performance liquid chromatography (UPLC) instrument (Waters Corporation, Milford, MA, USA) was employed for the separation and analysis of eluted glycans. This high throughput instrument generated a total plasma N-glycome chromatogram of 39 N-glycan peaks. Each glycan peak’s relative abundance was expressed as a percentage of the total integrated area (Fig. 2). Twenty-one (21) derived traits were calculated then calculated from the 39 N-glycan peaks (Supplementary Table 1).

### Statistical analysis

Batch correction and normalisation on the UPLC data was performed to control for non-biological variability. To explore batch effects, data tables were created for each plate that compared T2DM, age and gender. Glycome experiments were designed to control for important factors between the different plates. These factors (type II diabetes vs healthy control, male vs female, age separated at the median, timepoint of data collection) were evenly distributed among the experimental batches. During data processing, experimental artefacts were removed by using the ComBat method for batch correction. Thereafter, data was normalised using median quotient normalisation. Finally, all glycan data was rank transformed before analyses. Also, PCA was performed to check for clustering of plate samples. Variables that were positive for batch effects were adjusted according to the recommendations of Leek et al.^33^.

Kolmogorov Smirnoff test and QQ plots was viewed to ascertain where the data was normally distributed or not. Continuous data was represented as mean ± standard deviation (Mean ± SD) while categorical variables were expressed as frequencies (percentages). Groups comparisons for continuous variables performed either by Student-t tests or Mann–Whitney U-tests whereas categorical variables were compared using Chi-square tests. The Benjamini–Hochberg (BH) method was used to control the false discovery rate (q). Spearman correlational analysis was carried out to establish associations among the biochemically N-glycan measurements. Agglomerative hierarchical clustering was derived using the Euclidean distance as the similarity measure and Ward methodology. The dendrogram for the columns indicated four possible clusters for the biochemical measurements.

### Multivariate integrative framework

DIABLO extends the ideas of sparse generalized canonical correlation analysis (sGCCA). Let $X^{\left( 1 \right)} , \ldots ,X^{\left( J \right)}$ denote $J$ normalized, cantered, and scaled datasets of dimensions $\left( {N \times P_{1} } \right), \ldots ,\left( {N \times P_{J} } \right)$, measuring the expression levels of $P_{1} , \ldots ,P_{J}$ multi-block variables on the same sample $N$. sGCCA identifies relevant dimensions, $d = 1, \ldots , D,$ of the multi-block dataset by maximizing the variance–covariance function

$$\mathop {\max }\limits_{{a_{d}^{\left( 1 \right)} , \cdots ,a_{d}^{\left( J \right)} }} \mathop \sum \limits_{i,h = 1, i \ne h}^{J} c_{i,h} {\text{cov}}\left( {X_{d}^{\left( i \right)} a_{d}^{\left( i \right)} ,X_{d}^{\left( h \right)} a_{d}^{\left( h \right)} } \right),$$

$${\text{s}}.{\text{t}}.\left\| {a_{d}^{{\left( j \right)}} } \right\|_{2} = 1\;{\text{and}}\;\left\| {a_{d}^{{\left( j \right)}} } \right\|_{1} \le \lambda ^{{\left( j \right)}} \;for\;all\;1 \le j \le J$$

where $a_{d}^{\left( j \right)}$ is the loading vector on dimension $d$ associated with the residual matrix $X_{d}^{\left( j \right)}$ of the dataset $X^{\left( J \right)} .$ $C = \left\{ {c_{i,h} } \right\}_{i,h}$ is a $J \times J$ design matrix that indicates the connections among multi-block dataset. Elements in $C$ can be interpreted as correlations where zero indicates that the blocks of data are not connected, and one indicates that they are fully connected. $\lambda^{\left( j \right)}$ is a non-negative parameter that controls the amount of shrinkage, indicating the number of non-zero coefficients in $a_{d}^{\left( j \right)}$, for each component score $s_{d}^{\left( j \right)} = X_{d}^{\left( j \right)} a_{d}^{\left( j \right)}$.

The data was partitioned to create two separate sets of data, one for training the models and one for testing their predictive performance. This division occurred at an 80/20 proportion of the data. K-fold cross-validation (CV) was used to evaluate and compare the different models to each other. Analysis was performed in R statistical software. DIABLO was implemented in the ‘*mixOmics’* R Bioconductor package which has functions for parameters’ choice and visualization to assist in the interpretation of the integrative analyses.

## Results

The demographic and anthropometric information detailed in Table 1 shows that there were more female participants (61.4%), along with a mean age of controls and cases been 56.54 ± 9.89 and 55.10 ± 9.27, respectively. Majority of the participants in both groups had some form of education (χ^2^ = 9.83, q = 0.0812) and employment (χ^2^ = 26.74, q = 0.0003). BMI (q = 0.8262), TC (U = 21,918; q = 0.9604), LDL-c (U = 20,545; q = 0.3322), TG (U = 22,012, q = 0.9050) was not statistically different in both groups. Both HbA1c (U = 9768.3; q = 0.0001) and FBS (U = 9871.5; q = 0.0001) were high in T2DM compared to control but surprisingly, there was a higher SBP (U = 20,863.5, q = 0.0084) and a higher HDL-c (178.68, q = 0.0010) in controls and cases, respectively.

**Table 1:** Characteristics of participants with and without T2DM.

Variable	Control	Case	Statistic	*p*	q
Age (mean ± SD)	55.10 ± 9.27	56.54 ± 9.89	− 1.466^t^	0.0648	0.1102
**Age (years)**					
31–40 years	8 (3.7)	14(6.0)	8.57^	0.073	0.1128
41–50 years	70(32.0)	50(21.6			
51–60 years	83(37.9)	87(37.5)			
61–70 years	44(20.1)	63(27.2)			
71–80 years	14(6.4)	18(7.8)			
**Gender**					
Female	135 (61.4)	133 (57.30)			
**BMI (kg/m**^**2**^**)**			1.302^	0.729	0.8262
Underweight	11(5.0)	7(3)			
Normal	91(41.6)	102(44.2)			
Overweight	74(33.8)	77(33.0)			
Obese	43(19.6)	45(19.5)			
**Education**			9.838^	0.043	0.0812
Tertiary	29(13.3)	40(17.2)			
Senior high	72(33.0)	53(22.8)			
Junior high	71(32.6)	76(32.8)			
Lower primary	28(12.8)	28(12.1)			
No formal education	18(8.3)	35(15.1)			
**Occupation**			26.743^	0.0001*	0.0003**
Employed	147(67.4)	152(65.8)			
Retired	21(9.6)	27(11.7)			
Keeping house	14(6.4)	23(10.0)			
Unemployed	26(16.6)	29(12.5)			
**Physical activity**					
Sedentary	30(13.8)	53(22.9)	9.772^	0.021	0.0446**
Moderate activity	114(52.3)	94(40.7)			
Active	74(34.0)	84(36.3)			
**Clinical/biochemical data**					
WHtR	0.56 ± 0.08	0.56 ± 0.08	24057^u^	0.4933	0.599
SBP (mmHg)	145.96 ± 24.3	139.78 ± 24.91	20,863.5^u^	0.0035*	0.0084**
DBP (mmHg)	84.70 ± 14.42	82.52 ± 13.10	22652^u^	0.0925	0.131
FPG (mmol/l)	5.86 ± 0.95	9.24 ± 4.26	9871.5^u^	0.0000*	0.0001**
HbA1c (mmol)	5.45 ± 1.00	8.23 ± 2.09	9768.3^u^	0.0000*	0.0001**
TC (mmol/l)	4.69 ± 1.26	4.66 ± 1.26	21,918.5^u^	0.9604	0.9604
TG (mmol/l)	1.35 ± 0.97	1.24 ± 0.54	22,012.5^u^	0.8518	0.905
HDL-c (mmol/l)	1.24 ± 0.33	1.35 ± 0.33	17868^u^	0.0003*	0.0010**
LDL-c (mmol/l)	2.88 ± 1.05	2.74 ± 1.16	20,545.5^u^	0.254	0.3322

Data presented as Mean ± SD and n (%). ^χ^2^ test of independence, ^t^Student’s t-test, ^u^Mann Whitney U tests. Tests of significance were two tailed (*p < 0.05); **q < 0.05F significant after correction for FDR and are bold.

### Discriminating signature for N-glycans on T2DM and healthy controls

**Figure 3:**
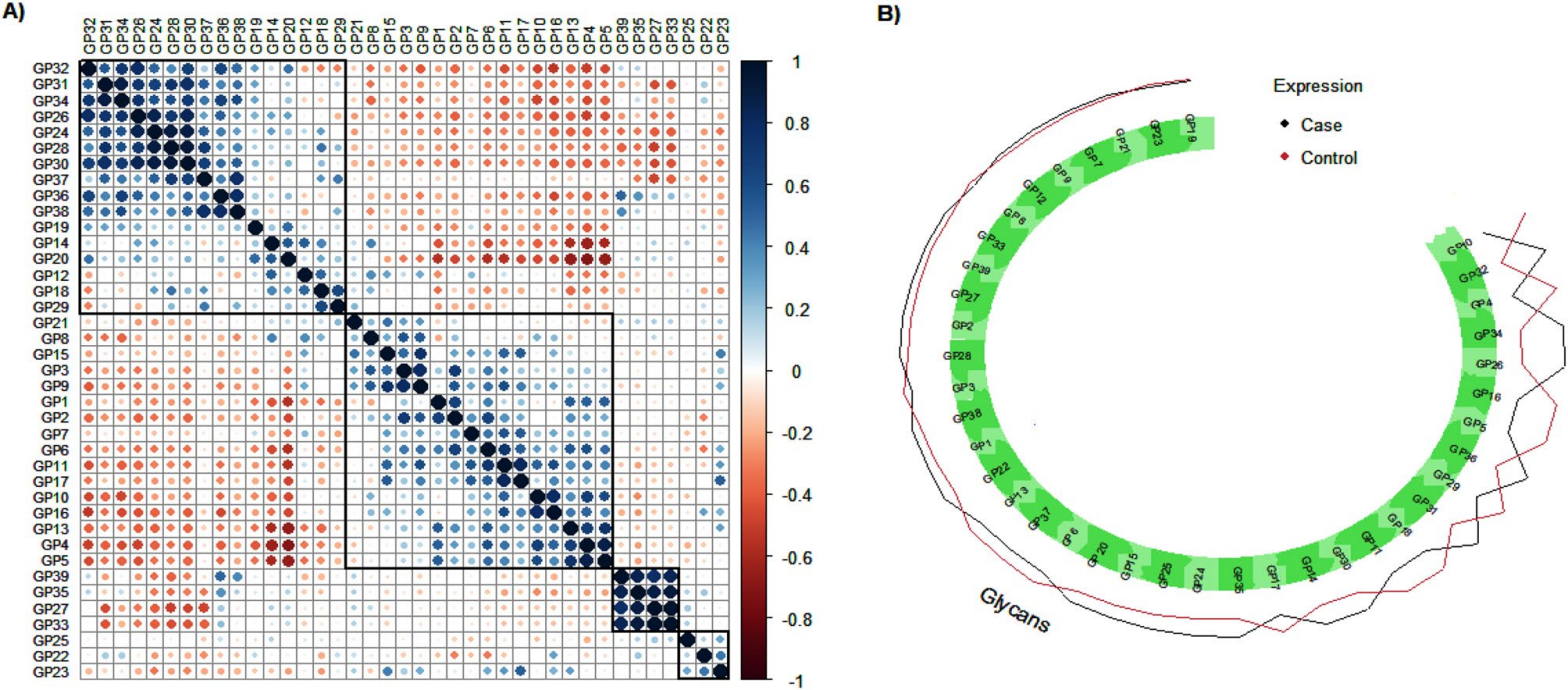
(**A**) Glycans correlation analysis for healthy controls and T2DM cases. The matrix presented are hierarchically clustered to highlight the signature of glycans expression in healthy controls and T2DM cases. (**B**) Expression of glycans in healthy controls and T2DM, ranked in terms of significant differing expression.

The nature of associations and patterns of clustering for the 39 N-glycans were explored for cases and controls. Hierarchical cluster analysis identified four clusters in the correlation of the expressive signatures of the 39 N-glycans across healthy controls and T2DM cases (Fig. 3A). For example, cluster A had 16 N-glycans (Table 2), of which 11 were found to express significantly different (Fig. 3B) between healthy controls and T2DM cases $(K = 16;k = 11,p < 0.05)$. Similarly, 16 glycans were clustered as B, of which 8 were found to express significantly different between healthy controls and T2DM cases $(K = 16;k = 8,p < 0.05)$. Cluster C and D had 4 and 3 glycans respectively and in each of them only 1 was found to express significantly different between healthy controls and T2DM cases (Table 2). No clear pattern was observed in how the clusters relate the canonically derived traits displayed in Supplementary Table 1.

**Table 2:** Analysis of N-glycans expressive signatures between T2DM cases and healthy controls across the four clusters.

Clusters	N-glycans	Univariate analysis
A	**GP 32**, **GP 31**, **GP 34**, **GP 26**, **GP 24**, GP 28, **GP 30**, GP 37, **GP 36**, GP 38, GP 19, **GP 14**, **GP 20**, GP 12, **GP 18**, **GP 29**	$K = 16;k = 11,p < 0.05$
B	GP 21, GP 8, **GP 15**, GP 3, GP 9, GP 1, GP 2, GP 7, **GP 6**, **GP 11**, **GP 17**, **GP 10**, **GP 16**, GP 13, **GP 4**, **GP 5**	$K = 16;k = 8,p < 0.05$
C	GP 39, **GP 35**, GP 27, GP 33	$K = 4;k = 1,p < 0.05$
D	**GP 25**, GP 22, GP 23	$K = 3;k = 1,p < 0.05$

NB: Bold text indicates the glycan expression is significantly different between T2DM cases and healthy controls, compared at 5% level of significance.

**Figure 4:**
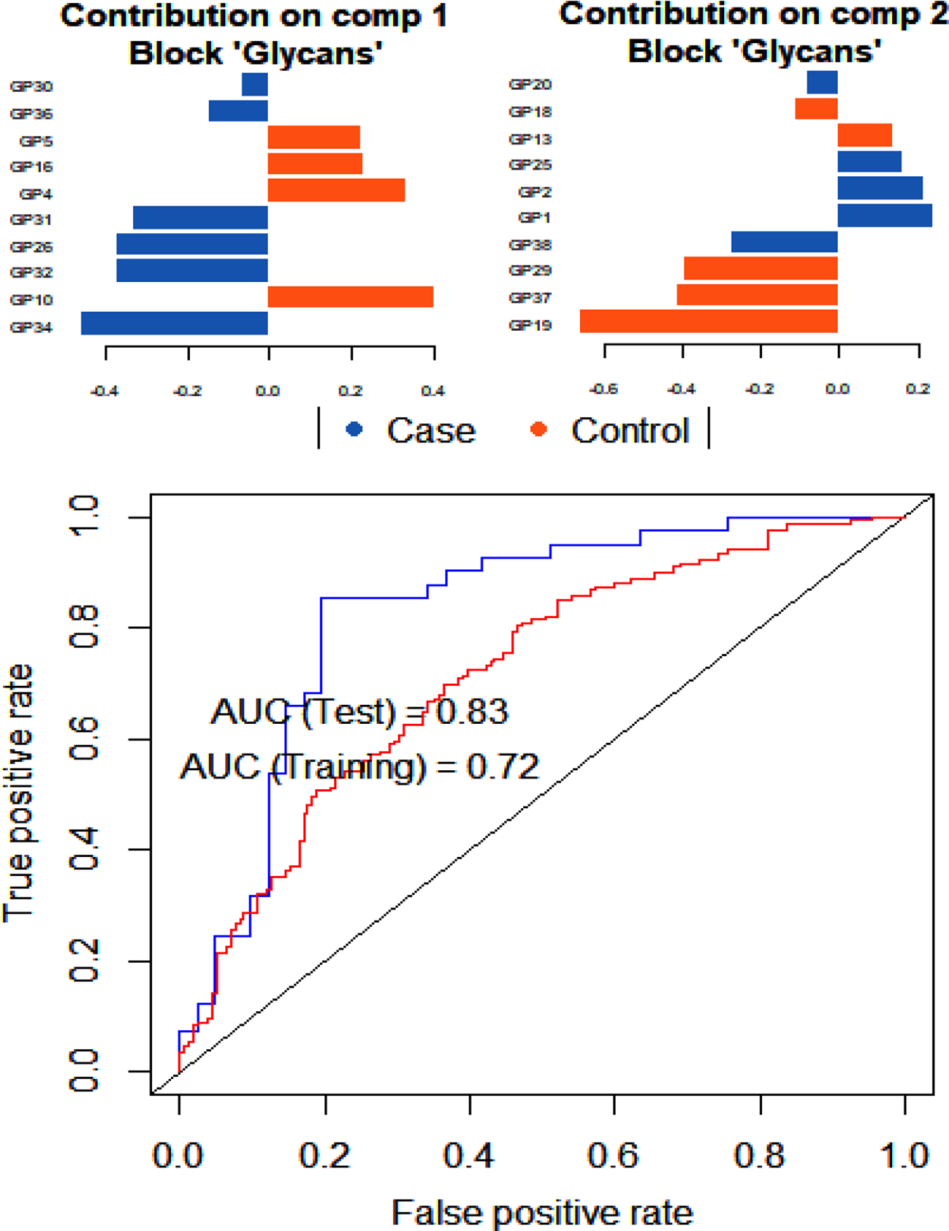
Principal component and discrimination analysis of top expressive glycans in T2DM and healthy controls. Feature selections are important in the refinement of biological and biochemical hypotheses. We identified a combination of discriminative features from a disparate block of N-glycan data set. N-glycan peaks loaded differently along the two principal components (PC), with estimates of positive and negative weights. A large absolute value indicates the importance of the variable to the PC and the colour codes indicate how prominent the biomarker expressed in T2DM and healthy controls. Selected variables were ranked from the least important to the most important. The classification accuracy the training and testing of the discriminant function formulated using the 10 glycans associated with component 1 reveals a model with good learning rate.

Feature selection is important in the refinement of biological and biochemical hypotheses. We identified a combination of discriminative features from a disparate block of glycans. N-glycans loaded differently along the two principal components (PC), with estimates of positive and negative weights (Fig. 4). A large absolute value indicates the importance of the variable to the PC and the colour codes indicate how prominent the biomarker expressed in the cases of Type II diabetes mellitus and healthy controls. To discriminate between T2DM and healthy controls, the optimal model identified the N-glycans signatures and expressed their contributions in classifying between T2DM and healthy controls for 10 out of 39 N-glycans in each component. The top 10 discriminatory glycans for each PC were ranked from the most important (top) to the least important. Sample plot of the final DIABLO model displayed a better discrimination of T2DM and healthy controls with plasma glucose measures compared (Fig. 4). For example, in component 1, GPs 34, 32, 26, 31, 36 and 30 were found to significantly express highly in T2DM, whereas GPs 10, 4, 16 and 5 were significantly expressed in the healthy controls. On the second principal axis, GPs 19, 37, 29, 13 and 18 were highly expressed in the healthy controls, whilst GPs 38, 1, 2, 25 and 20 were dominant in T2DM. Based on the results from the PCA, the 10 glycans that highly contributed to component 1, were used in a discriminant analysis. A data split of 80% and 20% were used for training and validation purposes. The area under the curve (AUC) for the training phase was 0.72, which improved to 0.83 for the test data, highlighting a good learning rate for the discriminant model.

### Multivariate integrative analysis

**Figure 5:**
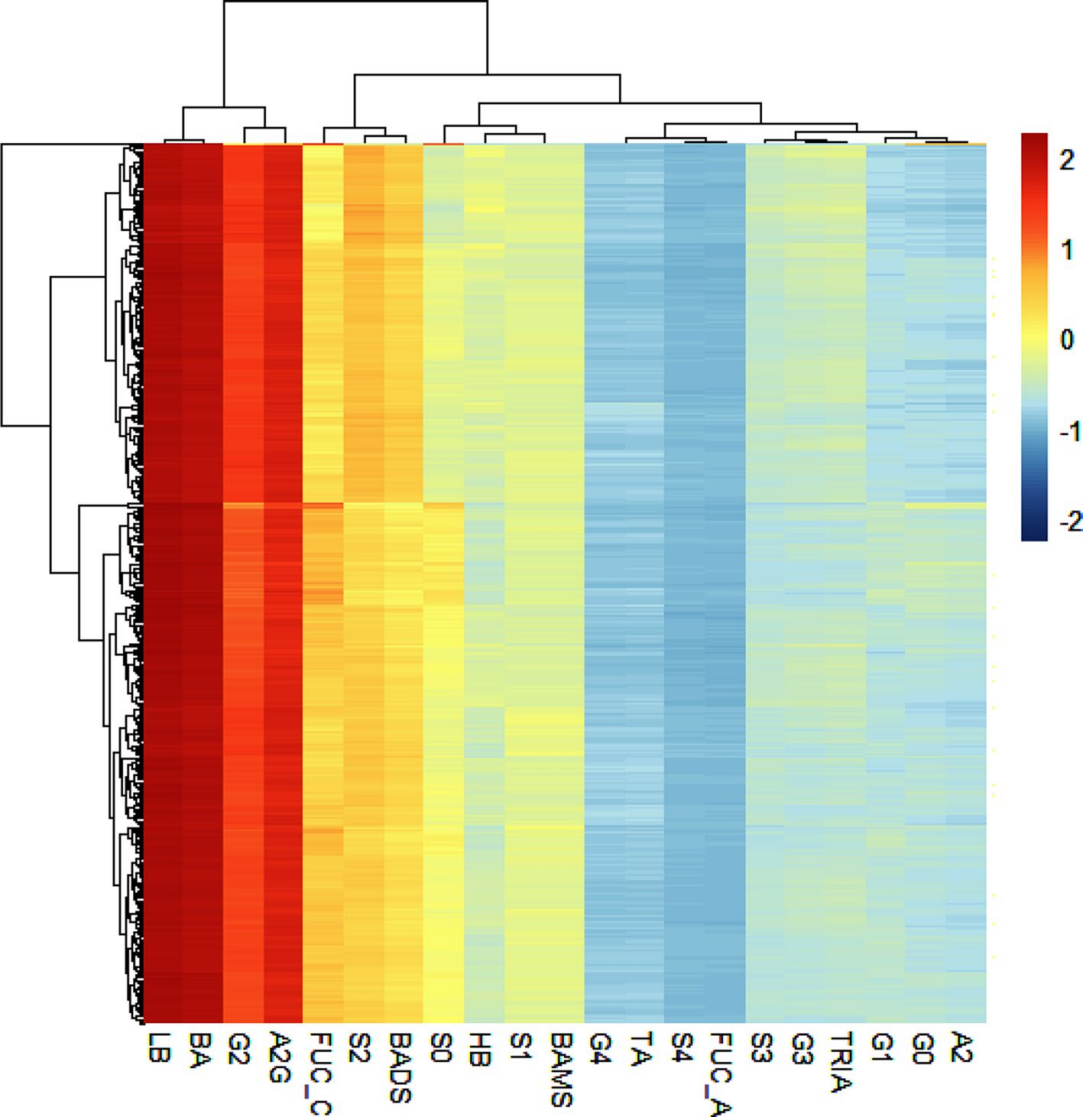
Hierarchical clustering of derived N-glycans traits in cases and controls. Hierarchical clustering of the cases and control samples using the measurement of sugars and lipids from block sPLSDA-reg network. Agglomerative hierarchical clustering was derived using the Euclidean distance as the similarity measure and Ward methodology. The red colour indicates that the row-column clusters are positively correlated, and the light blue colour indicates a negative correlation, whereas yellow indicate weaker correlation values.

We investigated the clustered image map (CIM) to highlight the strength and direction of pair-wise association structures between the two groups and the canonically derived traits. We then selected the important features between multi-block derived traits of the N-glycan measurements. CIM based on a hierarchical clustering simultaneously operated on the rows and columns using a similarity matrix to produce a 2-dimensional coloured image (Fig. 5). The dendrogram for the columns indicated six possible clusters for the canonically derived traits of biochemical N-glycans measurements, reflecting the six unique characterisations of branching, degree of branching, level of galactosylation, level of sialylation, sialylation of biantennary and position of fucose.

**Figure 6:**
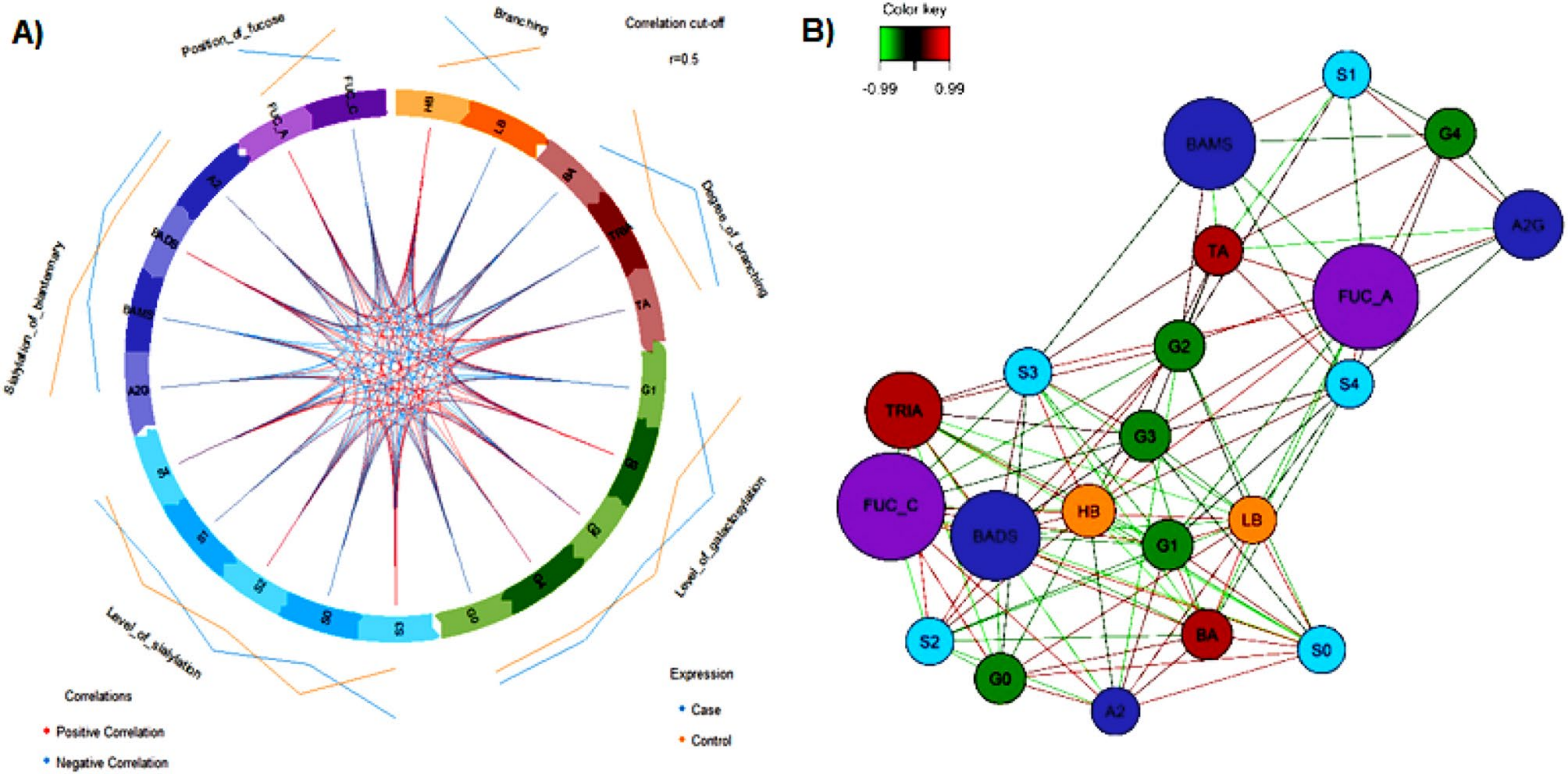
Correlation and relevance N-integrative supervised analysis with DIABLO. (**A**) Circos plots showing inter-block correlations. Spearman rank correlations were calculated for each pairwise comparison of variables. Variables with r = 0.5 between-block correlation were presented, (**B**) relevance network visualisation of the selected features.

Multi-block analysis of the canonically N-glycan derived traits is presented in a circos plot (Fig. 6A), with links between or within blocks indicating positive and negative correlations at a cut-off correlation of |0.5|. This threshold was arbitrarily chosen to obtain interpretable networks that were neither too sparse nor too dense. We observed significant difference of expression $(p < 0.05)$ of S1, BAMS, A2G and G2 being expressed highly in the healthy control group compared to T2DM cases, whereas G3, TRIA, HB and S3 expressed significantly highly in T2DM cases compared to healthy controls (Fig. 6A).

Using a pairwise similarity matrix directly obtained from outputs of sGCCA and PLS, bipartite network was inferred (Fig. 6B). One relevant component was obtained when the threshold was set to 0.5, linking the corresponding correlated subsets in the independent and dependent data. The network model representing the bivariate partial correlation matrix between the 21 canonically derived traits, comprises both positive (red lines) and negative (green lines) correlations (Fig. 6B). HB, G1 and G3 were central in the network and observed more connections than the others, highlighting their importance in discriminating between T2DM and healthy controls.

## Discussion

Comprehensive understanding of spectral N-glycan data from UPLC analysis is anchored on advanced statistical methods. Integrative methods offer comprehensive means to dissect data, with the goal of transforming the data into a clinically useful information. For the first time, we have applied a powerful and advanced integrative method DIABLO, to explore N-glycan profiles interaction in real time.

Prior to applying the DIABLO method, univariate, and multivariate statistical methods (e.g., student *t* tests and Mann Whitney U tests and chi-square test) have been used to reveal the association between T2DM and biochemical measures such as plasma glucose and lipid profiles. Surprisingly, the control group had a higher blood pressure than the cases, and this can be attributed to the medication use (glucose, lipid and blood pressure lowering drugs) among the cases (Table 1). Moreover, this highlights the proportion of the population who have raised blood pressure, but they are unaware of it. WHO reports that an estimated 45% of hypertensive adults are not aware of it, although the control group in the current study cannot be said to be hypertensive. This is because hypertension is established after repeated measures of blood pressure above normal threshold (140 mmHg). In the present study, blood pressure was only measured once. It is not clear why the control group had a lower HDL-c but it may be attributed to genetic factors or defects in cholesterol efflux.

Medication use in T2DM can potentially affect their N-glycome. Singh et al.^30^ found that statin use was linked to a decrease in all fucosylated traits including diantennary and triantennary structures (A2EF, A2LF, A3EF, A3L0F). In addition, statin use was associated with an increased galactosylation in diantennary non-fucosylated (A2F0G) and in sialylated diantennary (A2SG) glycans. The study further stated that statin use negatively correlated with Alpha2,6-sialylation of triantennary (A3E) and fucosylated tetra-antennary glycans (A4FGE). Similarly, metformin correlated with a decreased fucosylation in diantennary, triantennary and tetra-antennary traits and an increase of galactosylation in diantennary glycans^34^.It is widely known that T2DM develops several years before clinical diagnosis. Mild symptoms such as weight loss or weight gain, fatigue, increased hunger would progressively result in persistent high plasma glucose and complications. However, because of limited sensitive, and robust biomarkers, T2DM diagnosis is often delayed. This problem appears to be solved with the advent of N-glycans. First, the GST2D score was used to predict T2DM development 6–8 years before clinical manifestation^35^. In another study, Cvetko et al.^36^ reported that individuals who were healthy at baseline but developed insulin resistance and T2DM over time, were characterised by complex and highly branched N-glycan structures. Specifically, the study identified alterations in eight N-glycans: GP10, GP16, GP18, GP19, GP20, GP26, GP32 and GP34^36^; with GP 32 and GP34 being the most significant in the continuum of insulin resistance and T2DM. Increasing evidence shows that T2DM patients can be distinguished from healthy individuals depending on the composition of their respective total N-glycome^5,18^. Thus, we explored the N-glycan traits whose expression were different in cases and controls. The present study validates that of Cvetko et al.^36^ and Clemens et al.^35^, we identified GPs 34, 32, 26, 31, 36 and 30 to be highly expressed in T2DM in the first principal axis and on the second principal axis, GPs 38, 1, 2, 25 and 20 were dominant in T2DM. Sialylated glycans (GP26, GP32, GP35 and GP36) are expressed on a1-acid glycoprotein, whereas GP18 and GP20 originates from glycoproteins a-antitrypsin. A-antitrypsin is a protease inhibitor with at least three glycosylation sites for biantennary glycans without fucosylation (site asparagine 70), bi-, tri- and tetra-antennary glycans with core and antennary fucosylation (at site asparagine 107) and site asparagine 271 is occupied by bi- and tri-antennary glycans with core- and antennary-fucosylation^37^. A-antitrypsin protects β-cells from apoptosis and triggers insulin secretion, hence important for preventing type I diabetes^38^.

Clerc et al.^39^, further states that triantennary (GP 30, GP 31 and GP 32) and tetraantennary (e.g., GP 26, GP34, 36) glycans are expressed on kininogen-1 and histidine-rich glycoproteins. Kininogens are proteins with multiple functions including antidiuretic, antiangiogenic, antithrombotic, profibrinolytic and proinflammatory proteins. Abnormal expression of this glycoprotein is linked to diabetes^40^. Histidine-rich glycoproteins bind to ligands including heparan sulfate, plasminogen, heme amongst others and regulates multiple processes such as cell adhesion, fibrinolysis, cell chemotaxis. A deficiency of this protein has been associated with thrombosis, but its role in diabetes has also been reported^41^. Similarly, abnormal activities of a-antitrypsin, transferrin and hemopexin are all implicated in diabetes. Of particular interest is three glycan groups (GP30, GP36 and GP38) that have been shown to have clinical relevance in maturity onset diabetes of the young (MODY)^42^. In fact, Juszczak et al.^42^, documented that GP30, GP36 and GP38 had the best discriminative power between HNF1A-MODY and early-onset type 2 diabetes. The authors explained that HNF1A is a transcription factor for the inflammatory marker C-reactive protein (CRP) and a master regulator of fucosylation; with variations in HNF1A triggering MODY. With a sensitivity of 88% and specificity of 80%, was the best amongst the three glycan groups in discriminating between individuals with damaging HNF1A alleles from those with early-onset nonautoimmune diabetes but lacked HNF1A variants. The study showed that subjects with deleterious HNF1A allele had reduced levels of these glycans than those who lacked the rare HNF1A allele^42^.

The findings of the current study build upon that of Keser et al.^17^ who also suggested that the increased branched N-glycans in T2DM can be due to dysregulation of the hexosamine biosynthesis pathway (HBP). HBP has been found to be involved in the metabolism of glucose. This pathway under normal conditions, metabolises up to 3% glucose of the total glucose in the body. However, when homeostatic mechanism is disturbed, such as in T2DM, the metabolism of glucose is heightened, producing uridine diphosphate N-acetylglucosamine (UDP-G1cNAc). UDP-G1cNAc is a substrate for glycosyltransferases that catalyses the elongation and branching of glycan chains in glycosylation. GNTs are encoded by MGAT3 [mannosyl (β-1,4-)-glycoprotein β-1,4-N-acetylglucosaminyltransferase] but specifically, GNT-I, -II, -IV and -V catalyses the biosynthesis of mono, bi, tri and tetra-antennary glycans whereas GNT extends the 1–6 arm of the glycan core with GlcNAc residue. A defective GNT glycosyltransferase in the pancreatic islets results in impaired insulin action, impaired glucose tolerance and eventually, hyperglycaemia.

Aberration of fucosylation, be it core or antennary has been implicated in our results just as stated in multiple chronic diseases^43–45^. For example, Herrera et al.^46^ identified core-fucosylated tetra-antennary glycan to be associated with poor breast cancer prognosis. Then Testa et al.^44^, showed that core-α-1,6-fucosylated diantennary glycans was associated with T2DM. Sialic acids (N-acetylneuraminic acids) are pinned to the non-reducing ends of N-glycans by way of 2,3-, ,2,6- linkages. When bound, they play crucial roles in the pathological conditions including cancers and viral infections, while sialic acid complex glycans have been suggested to have anti-inflammatory properties^47^. Removal of UDP-N-GlcNAc 2-epimerase/ N-acetylmannosamine (ManNAc) kinase, an enzyme required for the biosynthesis sialic acids, led to glomerula proteinuria in mice^48^. In addition, other studies have found that upregulation of β-galactoside α-2,6-sialyltransferase 1, an enzyme that catalyses terminal α2,6-sialylation, was associated with worse patient outcomes in cancer^49^. Other studies have also indicated that an increase in α-(2 → 3)-sialic acid correlates with tumor metastasis. For example, intravenous administration of a sialidase (enzyme that cleaves sialic acids) blocking agent caused an increase release of insulin in pancreatic islets^50^. It is known that hyposialylated IgG glycans stimulates endothelial FcγRIIb, which has been previously associated with insulin resistance in obese mice. In the present study, the T2DM was associated with terminal sialylation. Recently, increased sialic acids on N-glycans has been implicated in T2DM development^17^. The absence of sialic acids on plasma LDL-c has been shown to induce cholesterol ester accumulation in cells and hence implicated in cardiometabolic diseases. This could be a possible reason why plasma LDL-c was highly loaded in cases compared to controls^51^.

The main limitation of the study relates to the small sample, and which means, the results cannot be generalised. Also, there is a possibility of biological variations related to gene expression in the samples, but that was not investigated. Already a genome wide association study has identified HNFA1 α as the master regulator of fucosylation^52^. Moreover, Cohain et al.^27^ analysis on cardiometabolic tissues revealed multiple genes that code for clinical markers including total cholesterol (DHCR7, FADS1, FADS2, MMAB, and MVK), (FLVCR1, LSS, MMAB, MVK, DHCR7, FADS1, FADS2 and VPS37D), LDL-c (FADS1, FADS2, and LSS), HDL-c (FLVCR1, MMAB, MVK, FADS1, FADS2), and TG (VPS37D, FADS1, FADS2). Zaytseva^53^ also reported that most of the highly heritable N-glycan peaks such as GP1, GP2, GP4-6, GP10-11, GP16, and GP17 were core-fucosylated biantennary with reduced sialylation whereas GP 20 and GP 14 had a low heritability. We intend to use path analysis and confirmatory factor analysis to determine gene-glycan relationships.

The present study has only provided information about glycans in biological samples (glycome), without highlighting downstream changes in the transcriptome, metabolome, lipidome and proteome. Thus, combining and analysing multiomics simultaneously will provide a clearer understanding of the mechanism that underly T2DM pathogenesis.

## Conclusion

DIABLO is a robust method that captures the N-glycan-glycan interactions in T2DM and healthy controls. T2DM is associated with highly branched N-glycan structures including trigalactosylated, triantennary, high branching (HB) and trisialylated that are derived from glycoproteins. Glycan groups identified to discriminate T2DM from healthy controls can be exploited further to unearth their potential for T2DM diagnosis and prognosis.

## Supplementary Information

Supplementary Information.
