# Author Correction: Multi-block data integration analysis for identifying and validating targeted N-glycans as biomarkers for type II diabetes mellitus

**DOI:** 10.1038/s41598-022-21099-2

**Published:** 2022-10-05

**Authors:** Eric Adua, Ebenezer Afrifa-Yamoah, Emmanuel Peprah-Yamoah, Enoch Odame Anto, Emmanuel Acheampong, Kwaafo Akoto Awuah-Mensah, Wei Wang

**Affiliations:** 1 Rural Clinical School, Medicine and Health, University of New South Wales, Sydney, NSW Australia; 2 School of Medical and Health Sciences, Edith Cowan University, Joondalup, WA Australia; 3 School of Science, Edith Cowan University, Joondalup, WA Australia; 4 Department of Chemistry, East Tennessee State University, Johnson City, TN USA; 5 Department of Medical Diagnostics, Faculty of Allied Health Science, Kwame Nkrumah University of Science and Technology, 9800 Kumasi, Ashanti Region Ghana; 6 Department of Mathematics, University of Environment and Sustainable Development, Somanya, Ghana; 7 Centre for Precision Health, Edith Cowan University, Joondalup, Australia

Correction to: *Scientific Reports* 10.1038/s41598-022-15172-z, published online 29 June 2022

The original version of this Article contained errors in Table 1, where values in column “Control”, under the subheading “Clinical/biochemical data” were omitted for TC (mmol/l), TG (mmol/l), HDL-c (mmol/l) and LDL-c (mmol/l). The complete and incomplete tables appear below.

Incomplete:

**Table:** 

Variable	Control
**Clinical/biochemical data**	
WHtR	0.56 ± 0.08
SBP (mmHg)	145.96 ± 24.3
DBP (mmHg)	84.70 ± 14.42
FPG (mmol/l)	5.86 ± 0.95
HbA1c (mmol)	5.45 ± 1.00
TC (mmol/l)	
TG (mmol/l)	
HDL-c (mmol/l)	
LDL-c (mmol/l)	

Complete:

**Table:** 

Variable	Control
**Clinical/biochemical data**	
WHtR	0.56 ± 0.08
SBP (mmHg)	145.96 ± 24.3
DBP (mmHg)	84.70 ± 14.42
FPG (mmol/l)	5.86 ± 0.95
HbA1c (mmol)	5.45 ± 1.00
TC (mmol/l)	4.69 ± 1.26
TG (mmol/l)	1.35 ± 0.97
HDL-c (mmol/l)	1.24 ± 0.33
LDL-c (mmol/l)	2.88 ± 1.05

The original Article has been corrected.

