# Supplementary material for: Multi-block data integration analysis for identifying and validating targeted N-glycans as biomarkers for type II diabetes mellitus
Source: Sci Rep. 2022 Jun 29;12:10974. doi: 10.1038/s41598-022-15172-z (PMC9243128; doi:10.1038/s41598-022-15172-z)
Supplement: Supplementary file 1 — Supplementary Information. [file 41598_2022_15172_MOESM1_ESM.docx]

**Supplementary table 1**

| **Variable** | **Label** | **Description** |
| --- | --- | --- |
| LB | Low Branching | GP1+GP2+GP3+GP4+GP5+GP6+GP7+GP8+GP9+GP10+GP11+GP12+GP13+GP14+GP15  +GP16+GP17+GP18+GP19+GP20+GP21+GP22+GP23 |
| HB | High Branching | GP24+GP26+GP27+GP28+GP29+GP30+GP31+GP32+GP33+GP34+GP35+  GP36+GP37+GP38+GP39 |
| S0 | Neutral | GP1+GP2+GP3+GP4+GP5+GP6+GP7+GP8+GP9+GP10+GP11 |
| S1 | Monosialylated | GP12+GP13+GP14+GP15+GP16+GP17 |
| S2 | Disialylated | GP18+M9+GP20.21+GP22+GP23+GP24++ GP25+GP26 |
| S3 | Trisialylated | GP27+GP28+GP29+GP30+GP31+GP32+GP33+GP34+GP35 |
| S4 | Tetrasialylated | GP36+GP37+GP38+GP39 |
| G0 | Agalactosylated | GP1+GP2+GP7 |
| G1 | Monogalactosylated | GP3+GP4+GP5+GP6+GP12+GP13 |
| G2 | Digalactosylated | GP8+GP9+GP10+GP11+GP14+GP15+GP16+GP17+GP18+M9+GP20+GP21+GP22+GP23 |
| G3 | Trigalactosylated | GP24+GP26+GP27+GP28+GP29+GP30+GP31+GP32+GP35 |
| G4 | Tetragalactosylated | GP33+GP34+GP36+GP37+GP38+GP39 |
| FUC_A | Antennary Fucose | GP32+GP35+GP39 |
| FUC_C | Core Fucose | GP1+GP2+GP4+GP5+GP6+GP10+GP11+GP13+GP16+GP17  +GP22+GP23+GP29+GP31 |
| BA | Biantennary | GP1+GP2+GP3+GP4+GP5+GP6+GP8+GP9+GP10+GP11+GP12+GP13+  GP14+GP15+GP16+GP17+GP18+GP20+GP21+GP22+GP23 |
| A2 | Biantennary Agalactosylated | GP1+GP2 |
| A2G | Biantennary Galactosylated | GP3+GP4+GP5+GP6+GP8+GP9+GP10+GP11+GP12+GP13+GP14+GP15  +GP16+GP17+GP18+GP20+GP21+GP22+GP23 |
| BAMS | Monosialylated Biantennary | GP12+GP13+GP14+GP15+GP16+GP17 |
| BADS | Disialylated Biantennary | GP18+GP20+GP21+GP22+GP23 |
| TRIA | Triantennary | GP19+GP24+GP26+GP27+GP28+GP29+GP30+GP31+GP32 |
| TA | Tetraantennary | GP33+GP34+GP35+GP36+GP37+GP38+GP39 |

This high throughput instrument generated a total plasma N-glycome chromatogram of **39** N-glycan peaks (GP). Each glycan peak’s relative abundance was expressed as a percentage of the total integrated area (Figure 2). Twenty-one (21) derived traits were calculated then calculated from the 39 N-glycan peaks.
